# Human immunodeficiency virus infection predictors and genetic diversity of hepatitis B virus and hepatitis C virus co-infections among drug users in three major Kenyan cities

**DOI:** 10.4102/sajhivmed.v19i1.737

**Published:** 2018-03-27

**Authors:** Micah Oyaro, John Wylie, Chien-Yu Chen, Raphael O. Ondondo, Anna Kramvis

**Affiliations:** 1 Immunology Unit, Department of Human Pathology, University of Nairobi, Kenya; 2 Department of Medical Microbiology, University of Manitoba, Canada; 3 Hepatitis Virus Diversity Research Unit (HVDRU), Department of Internal Medicine, School of Clinical Medicine, Faculty of Health Sciences, University of the Witwatersrand, South Africa; 4 Department of Medical Laboratory Sciences, Masinde Muliro University of Science and Technology, Kenya; 5 Kenya Medical Research Institute, Centre for Microbiology Research, Nairobi, Kenya

## Abstract

**Background:**

Drug users act as reservoirs and transmission channels for hepatitis B virus (HBV), hepatitis C virus (HCV) and human immunodeficiency virus (HIV) infections to the general population worldwide. Periodic epidemiological studies to monitor the prevalence and genetic diversity of these infections to inform on interventions are limited.

**Objective of the study:**

The objective of this study was to determine the predictors of HIV infection and genetic diversity of HBV and HCV among drug users in Kenya.

**Materials and methods:**

A cross-sectional study on previous drug use history among drug users was conducted in three Kenyan cities using a respondent-driven sampling method between January 2011 and September 2012. Blood samples were collected and analysed for the presence of HBV, HCV and HIV serological markers and to determine the genotypes of HBV and HCV.

**Results:**

The overall prevalence of HBV, HCV and HIV among drug users was 4.3%, 6.5% and 11.1%, respectively, with evidence of HBV/HIV, HCV/HIV and HBV/HCV/HIV co-infections. The HBV circulating genotypes were A1 (69%) and D6 (19%), whereas HCV genotypes were 1a (72%) and 4a (22%). Injection drug use was a significant predictor of HIV/HCV infections. Younger age (30 years; aOR (adjusted odds ratio) = 0.50, 95% CI (confidence interval): 0.33–0.76; *p* < 0.001) and early sexual debut (aOR = 0.54, 95% CI: 0.40–0.82; *p* < 0.05) were negatively associated with detection of any of the three infections. Injecting drug use was positively associated with HCV infection (aOR = 5.37, 95% CI: 2.61–11.06; *p* < 0.001).

**Conclusion:**

This high level of genetic diversity exhibited by HBV and HCV isolates requires urgent implementation of harm reduction strategies and continuous monitoring for effective management of the patients.

## Introduction

Substance abuse is a growing phenomenon and infection with blood-borne pathogens among drug users is a great problem around the world. Human immunodeficiency virus (HIV), hepatitis B virus (HBV) and hepatitis C virus (HCV) are common infections among drug users – both injecting drug users (IDUs) and non-injecting drug users (NIDUs).^1,2^ Being blood-borne, HBV, HCV and HIV infections are commonly transmitted through unsafe drug injection practices and sexual contact.^3^ Both HBV and HCV have been implicated in acute and chronic liver disease, with a rapid progression to liver cirrhosis and hepatocellular carcinoma.^4,5^ Currently, over 2 billion people worldwide are infected with HBV and about 240 million are chronic carriers.^6^ Approximately 780 000 deaths occur annually as a result of HBV infection, with an estimated 4.5 million new HBV infections occurring each year.^7^ An estimated 170 million people are infected with HCV worldwide with more than 350 000 deaths annually.^8^ Approximately, 26 million of the 37 million HIV-positive individuals in the world reside in Sub-Saharan Africa,^9^ highlighting the disproportionate impact this pathogen is having on this region of the world, which includes Kenya. Within the East African countries, Uganda has an HIV prevalence of 7.3% with 1.6 million people living with HIV.^10^ Tanzania has an HIV prevalence of 4.7% with an estimated 1.4 million people living with HIV^11^ while in Kenya, an HIV prevalence of 5.9% with an estimated 1.5 million people living with HIV has been documented.^11^ Previous data have shown HBV prevalence of 10%, 13.3% and 8.8% in Uganda, Kenya and Tanzania, respectively.^12,13,14^ Studies on HCV prevalence from Kenya, Uganda and Tanzania have shown a prevalence of 1.8%, 2.6% and 1.2%, respectively.^15,16,17^

Drug abuse in Kenya drew national interest in the early 1990s, with the emergence of the Kenyan HIV epidemic, and the phenomenon continues to increase faster in Kenya, as well as among other East African countries.^18^ Although substance abuse is widespread across major towns in Kenya,^19^ the practice is more common in the cosmopolitan coastal regions as a result of tourism, illicit drug trafficking and cultural practices.^20,21^

The majority of people who inject drugs in Kenya are concentrated in Nairobi and Mombasa^22^, although evidence of IDU has also been documented from Kisumu.^23^ The same practice has been reported from other East African major cities, which include Mwanza^24^ and Dar es Salaam in Tanzania,^25^ Zanzibar^26^ and Mauritius.^27^

Although HBV, HCV and HIV viral infections are considered to be endemic in Africa, the prevalence of HCV/HIV and HBV/HIV co-infections, as well as mono-infections, varies depending on the risk factors, individual behaviours, socio-demographic profiles and HBV immunisation coverage. A study conducted in two informal urban settlements in Nairobi, Kenya, showed HBV, HCV and HIV prevalence of 13.3%, 0.76% and 20.4%, respectively.^14^ Anti-HCV prevalence among blood donors in Nairobi was 1.8%,^15^ whereas among outpatients from three hospitals, an HBV surface antigen (HBsAg) positivity of 11.4% was documented.^28^ An HIV prevalence of 18.7% was reported among IDUs in Nairobi^7^ and 87.5% among injecting heroin users at the Kenyan coast,^29^ whereas an HCV prevalence of 16.4% and HCV/HIV co-infection prevalence of 17.9% among the same heroin users was documented.^29^

Hepatitis B virus belongs to the family *Hepadnaviridae* with a genome sequence of 3200 nucleotides in length.^30^ The partially double-stranded DNA has four overlapping open reading frames encoding the S (surface), C (core), P (polymerase) and X genes. The virus has a high mutation rate because the reverse transcriptase lacks proofreading ability with an estimated nucleotide substitution rate of 10^−3^–10^−6^/site per year.^31^ This error-prone replication accounts for point mutations, insertions and deletions found in HBV genotypes/subgenotypes.^32^ HBV is classified into nine genotypes (A–I) and a putative genotype, ‘J’.^33^ Depending on intergroup nucleotide divergence, HBV genotypes have been further classified into at least 35 subgenotypes.^34,35^ The global distribution of HBV genotypes and subgenotypes has been reviewed and shows distinct distribution, diversity and ability to adapt to different hosts.^35^ Within the African continent, HBV genotype A is confined to eastern, central and southern Africa, whereas genotype D prevails in northern countries and genotype E in western and central Africa. In Kenya, subgenotypes A1, D6 and D/E recombinant have been identified.^36,37,38^

Similarly, HCV exhibits a high degree of genetic diversity, which can create challenges in immune control and therapeutic intervention.^39^ HCV strains have been classified into seven genotypes and 67 subgenotypes.^40^ HCV genotypes differ at 30% – 35% of nucleotide sites whereas subtypes, strains of the same genotype, differ at < 15% nucleotide sites (43). Subtypes 1a, 1b, 2a and 3a have a global distribution.^41,42,43^ Genotypes 1, 2, 4 and 5 have endemic origins in Africa, with genotypes 1 and 2 occurring throughout Africa, genotype 4 in Egypt and parts of Central Africa and genotype 5 in southern Africa, and less commonly in some parts of central Africa.^44^

Two studies have been conducted to determine the prevalence, genetic diversity and distribution of HBV and HCV among drug users in Kenya. One of these studies found A1 as the dominant HBV genotype circulating among HIV-infected and HIV-uninfected drug users in coastal Kenya^45^ while the laboratory-based study among drug users from Nairobi found HCV genotypes 1a (73%) and 4 (27%).^46^ More information on the epidemiology and genetic distribution of HBV and HCV genotypes among drug users from major towns in Kenya to inform on appropriate interventions is needed. The objective was to determine the prevalence and genetic distribution of these viruses among drug users in Nairobi, Mombasa and Kisumu, towns in Kenya.

## Materials and methods

### Study population and data collection

A cross-sectional study among drug users (both injectors and non-injectors) on their previous drug use history using respondent-driven sampling (RDS) was conducted between January 2011 and September 2012. Through community seminars and RDS techniques, drug users from communities in Nairobi, Mombasa and Kisumu, cities in Kenya, were recruited. A group of trained nurse counsellors, community mobilisers and former drug addicts, with prior working experience on drug abuse cohorts, were employed to facilitate recruitment of participants. Male and female participants, aged 18 years or older, self-reported drug users and/or who had engaged in sex-related or drug-related HIV risk behaviours at least once monthly for three months prior to recruitment and willing to provide blood were eligible for recruitment into the study. The venues for recruitment were agreed upon by the group leader or representative. The initial potential participants from each city chosen by recruiters were referred to as ‘seeds’ and were provided with a coupon that they used to invite other members from their networks, whom they considered as potential participants for the study. Interviewers obtained signed informed consent from participants. All participants were able to read and understand the questionnaire in English without any translation. Participants who gave their consent were administered a quantitative questionnaire, counselled and blood specimens obtained. The questionnaire captured data on social demographic characteristics, risk factors and previous history on substance-use behaviours. Pre-and post-test counselling for HIV and other blood-borne infections (HBV/HCV) were offered as a package in the study. Participants were reimbursed with KSH 350 (US$ 7) for their time. The study was reviewed and approved by both Kenyatta National Hospital Ethics Research Committee (KNH/UON-ERC), approval # P144/5/2010, and University of Manitoba Health Research Ethics Board (HREB), approval # HS10896 (H2010:100), before it was conducted. The molecular testing of de-identified blood specimens for HBV and HCV was also approved by the Human Ethics Committee of the University of the Witwatersrand.

A total of 20 mL of venous blood (10 mL in EDTA tubes and 10 mL in plain tubes) was obtained from eligible individuals. Blood specimens were centrifuged and separated into plasma and serum, respectively, before being aliquoted into two parts and stored frozen at −80°C until analysed to determine prevalence of HIV, HBV and HCV, and identification of hepatitis B and C viral genotypes. Participants who tested positive for HIV, HBV and HCV were referred to clinical centres for treatment and further follow-up.

All participants were well informed about the study and their participation was kept confidential through a written consent and unique identifiers given to their samples, questionnaires and laboratory request forms including personal data. Descriptive statistics were presented as proportions and prevalence presented as % point estimate with its 95% confidence interval (CI). Differences in categorical variables were tested using Chi square and odds ratio (OR) for binary variables reported at 95% significance level (*p* < 0.05) using Stata version 14 (StataCorp 4905 Lakeway Drive, College Station, Texas 77845 USA)

### Laboratory testing procedures

#### Human immunodeficiency virus, hepatitis B virus and hepatitis C virus serology

Specimens were screened for HBV, HCV and HIV infection using HepanostikaHBsAgUltra^TM^ enzyme-linked immunosorbent assay (ELISA) (Biomerieux, France), AxSYM HCV (Abbott, Mississauga, ON) and AxSYM HIV1/2 gO (Abbott, Mississagua, ON), respectively.

#### Hepatitis B virus viral DNA isolation, amplification and sequencing

**Hepatitis B virus DNA extraction:** Hepatitis B virus DNA was extracted from the plasma samples using the *QIAampDNABlood Mini Kit* (QIAGEN GmbH, Hilden, Germany) according to the manufacturer’s instructions, and eluted in 100 µL of elution buffer. Known positive, negative sera and best-quality water were included as controls for the extraction.

**Hepatitis B virus amplification:** The basic core promoter/precore (BCP/PreC) region and complete S open read frame were individually amplified from the extracted specimen using nested polymerase chain reaction (PCR) primer sets.^47^ PCR reactions were carried out in the MyCycler™ thermocycler (Bio-Rad, Hercules, CA, USA). BCP/PreC PCR amplified from the nt 1606 to nt 1974 (from *Eco*RI site) by using BCP1 and BCP2 primer sets with PromegaTaq DNA polymerase (Promega, Madison, WI, USA). The complete S open reading frame (nt 2451 to 1254 from *Eco*RI site) was amplified using S1F/S1R and S2F/S2R primer sets together with HotStarTaq *Master Mix (*QIAGEN GmbH, Hilden, Germany).

Hepatitis B virus serology-negative specimens that did not amplify in the complete S region were subjected to the nested PCR to amplify the target surface region (nt 256 to nt 796 from *Eco*RI site). The amplicons were amplified using primer set 230F/800R for the first-round PCR,^48^ and primer set P7/P8 for second-round PCR^49^ together with PromegaTaq DNA polymerase (Promega, Madison, WI, USA). Amplicons were genotyped by restriction fragment length polymorphism (RFLP) analysis using *Hinf *I and *Tsp*509I.^49^

**Hepatitis B virus sequencing:** Amplicons were prepared for direct sequencing using HBV-specific primers and the BigDye Terminator v3.0 Cycle Sequencing Ready Reaction Kit (Applied Biosystems., Foster City, CA, USA). Sequencing was performed by the Central Analytical Facility, Stellenbosch University, South Africa, using the ABI 3130XL Genetic analyser (Applied Biosystems, Foster City, CA, USA). BCP/PreC sequences were analysed in the forward direction of a single fragment, while the complete S sequences of three overlapping fragments were analysed. The genotype or subgenotype could not be assigned in samples that failed to amplify in both the BCP and S region whereas those that amplified in either or both regions could be genotyped or subgenotyped

**Hepatitis B virus viral load quantification:** Hepatitis B virus viral load was quantified by real-time quantitative PCR using HBV-Taq1 and HBV-Taq2 primer sets together with the FAM/TAMRA labelled TaqMan BS-1 probe as previously described.^50,51^ The PCR reaction was performed using the ABI Prism 7500 (Applied Biosystems, Framingham, MA, USA).

Serial dilutions of the plasmid encoding a single genome of HBV DNA ranging from 10^2^ IU/mL to 10^9^ IU/mL were used to generate a linear standard curve. The second World Health Organization (WHO) International Standard for HBV Nucleic Acid Amplification Techniques, product code 97/750, with the final concentration of 1 IU/mL × 10^6^ IU/mL, was obtained from the National Institute for Biological Standards and Controls (NIBSC; Hertfordshire, UK). It was used as an internal positive control to calibrate and align the standard curve. The standard curve, blank, positive and negative controls and samples were all tested in duplicate.

### Hepatitis C virus viral ribonucleic acid isolation, amplification and sequencing

#### Hepatitis C virus RNA extraction and cDNA synthesis

Hepatitis C virus RNA was extracted from 38 out of 42 (90%) HCV serology-positive specimens, using the QiaAmp RNA mini kit (QIAGEN GmbH, Hilden, Germany), according to the manufacturer’s instructions. Known positive and negative sera and best-quality water were used as controls for the extraction procedure. HCV RNA was eluted with 60 µL elution buffer provided by the kit with the addition of RNase inhibitor (at final concentration of 1U/µL). First-strand HCV cDNA was synthesised immediately after extraction using the Superscript III First-strand synthesis system for RT-PCR (Invitrogen, Carlsbad, CA, USA) using the random hexamer at 100 ng per reaction.

#### Hepatitis C virus amplification

5’untranslated region (5’UTR), for HCV genotyping, and NS5B region, for HCV sub-typing, were amplified individually from the cDNA using nested PCR. Known HCV negative, positive sera best-quality water together with full length HCV Strain H77 (genotype 1a), pCNJ4C6S (HCV genotype 1b) and pJ6CF2a (HCV genotype 2a) HCV plasmid clones (obtained from Prof R. Purcell, NIH, USA) were included as controls.

5’untranslated region was amplified using published primers.^52^ Nested PCRs were carried out using Hotstar master mix (QIAGEN GmbH, Hilden, Germany) using 4 µL cDNA as template with forward and reverse primers at 1 µM final concentration in a final volume of 25 µL PCR mix. The cycling conditions were: an initial activation at 95°C for 15 min, followed by 40 cycles of denaturation at 94°C for 1 min, annealing at 50°C for 1 min, extension at 72°C for 1.5 min, followed by final extension at 72°C for 10 min. The second-round PCR used the same cycling condition as the first-round PCR.

NS5B region was amplified using published primers.^53^ Nested PCRs were carried out using Hotstar master mix (QIAGEN GmbH, Hilden, Germany) using 4 µL cDNA as template with forward and reverse primers at 1 µM final concentration in a final volume of 25 µL PCR mix. The cycling profile for the NS5B region was: an initial activation at 95°C for 15 min, followed by 40 cycles of denaturation at 94°C for 1 min, annealing at 60°C for 1 min, extension at 72°C for 1 min, followed by final extension at 72°C for 5 min. The second-round PCR used the same cycling conditions as the first-round PCR.

#### Hepatitis C virus sequencing

The amplicons were prepared for direct sequencing using the BigDye Terminator v3.0 Cycle Sequencing Ready Reaction Kit (Applied Biosystems., Foster City, USA) using second-round forward PCR primers. Sequencing was performed by the Central Analytical Facility, Stellenbosch University, South Africa, using the ABI 3130XL Genetic analyser (Applied Biosystems, Foster City, CA). 5’UTR and NS5B region sequences were analysed in the forward directions of a single fragment.

#### Ethical consideration

The study was reviewed and approved by both Kenyatta National Hospital University of Nairobi Ethics and Research Committee (KNH/UON-ERC) (approval # P144/5/2010) and University of Manitoba Health Research Ethics Board (HREB) (approval # HS10896) (H2010:100), before it was conducted. The molecular testing of de-identified blood specimens for HBV and HCV was also approved by the Human Ethics Committee of the University of the Witwatersrand.

## Results

Out of the 673 drug users enrolled, 626 (93%) were males, 74% (501/673) were NIDUs, while the remaining 26% were IDUs. Majority of the participants were aged between 20 and 34 years. Almost half of the participants, 304 (49.8%), had their main source of income from regular work, 158 (25.9%) from friends, and 128 (21%) from self-employment. None of the major tribes in Kenya (Kikuyu, Luyia and Luo) had a majority representation, indicating a mixed society when all participants were analysed together. Almost equal proportion of participants either stayed in rented houses (250 [40.1%]) or in a family member’s house (279 [44.7%]).

Overall, the prevalence was 11.1% (95% CI: 8.7 – 13.5) for HIV, 4.3% (95% CI: 2.8 – 5.8) for HBV and 6.5% (95% CI: 4.64 – 8.4) for HCV. The prevalence of HIV, HBV and HCV in men was 10.5% (95% CI: 8.1 – 12.9), 4.5% (95% CI: 2.9 – 6.1) and 6.6% (95% CI: 4.7 – 8.6), respectively, whereas for women it was 19.1% (95% CI: 7.9 – 30.3), 2.1% (95% CI: 0.38 – 11.12) and 6.4% (95% CI: 2.19 – 17.16), respectively (Table 1). Among the 23 samples with HBV mono-infections, 12 samples (52%) were from Nairobi, seven samples (30%) from Mombasa and four samples (17%) from Kisumu. Similarly, 27 samples had HCV mono-infections; out of these, 25 samples (93%) were from Mombasa, whereas two samples (7%) were from Nairobi. None of the samples collected from Kisumu participants tested positive for HCV mono-infection.

**TABLE 1:** Participant demographics and disease prevalence among drug users in three major cities in Kenya.

Participant characteristics	Variable	Male (*n* = 626)			Female (*n* = 47)			Total (*n* = 673)			*p*
		*n*	%	95% CI	*n*	%	95% CI	*n*	%	95% CI	
Age (years)	15–19	31	5.0	-	6	12.8	-	37	5.5	-	0.037
	20–24	223	35.6	-	18	38.3	-	241	35.8	-	0.712
	25–29	144	23.0	-	6	12.8	-	150	22.3	-	0.104
	30–34	130	20.8	-	6	12.8	-	136	20.2	-	0.188
	>34	98	15.7	-	11	23.4	-	109	16.2	-	0.164
Education level	Primary school level	392	62.6	-	29	61.7	-	421	62.5	-	0.900
	Secondary school level	187	30.2	-	17	36.2	-	204	30.6	-	0.365
	Tertiary level	40	6.4	-	1	2.1	-	41	6.1	-	0.239
Main source of income	Regular employment	304	49.8	-	24	52.2	-	328	50.0	-	0.013
	Friends and relatives	158	25.9	-	8	17.4	-	166	25.3	-	0.208
	Self employed	128	21.0	-	12	26.1	-	140	21.3	-	0.408
	Other	20	3.3	-	2	4.3	-	22	3.4	-	0.694
Ethnic group	Luo	115	19.4	-	7	14.9	-	122	19.1	-	0.036
	Luyia	63	10.6	-	5	10.6	-	68	19.6	-	0.900
	Kikuyu	43	7.3	-	7	14.9	-	50	7.8	-	0.043
	Kamba	33	5.6	-	5	10.6	-	38	5.9	-	0.124
	Other	339	54.2	-	23	49.0	-	362	53.8	-	0.489
Place lived most in the last 6 months	Own house	250	40.1	-	16	34.0	-	266	39.6	-	0.426
	Family member’s house	279	44.7	-	22	46.8	-	301	44.9	-	0.766
	Friend’s house	63	10.1	-	7	14.9	-	70	10.4	-	0.296
	Other	32	5.1	-	2	4.3	-	34	5.1	-	0.796
Infection Prevalence	HIV (*n* = 673)	66	10.5	8.1, 12.9	9	19.1	7.86, 30.34	75	11.1	8.73, 13.47	-
	HBV (*n* = 673)	28	4.5	2.88, 6.12	1	2.1	0.38, 11.12	29	4.3	2.77, 5.83	-
	HCV (*n* = 672)	41	6.6	4.65, 8.55	3	6.4	2.19, 17.16	44	6.5	4.64, 8.36	-

CI, confidence interval; HIV, human immunodeficiency virus; HCV, hepatitis C virus; HBV, hepatitis B virus.

Mono-infections were detected in 99 drug users, of whom 16 (16.2%) were IDUs, 72 (72.7%) were NIDUs and 11 (11.1%) were unclassified drug users. IDU was significantly associated with mono-infection among those detected with any of the three infections (OR = 4.5 [95% CI: 1.66–12.54], *p* < 0.01). Young age (30 years old) was not associated with mono-infection (OR = 1.91 [95% CI: 0.73–5-04], *p* = 0.22). Dual infections were detected in 22 drug users (10-IDUs, 8-NIDUs and 4-unclassified). Regarding HIV and hepatitis co-infections, HIV/HCV co-infections were observed in 18 participants; 13 (72%) were IDUs and five (28%) were NIDUs. HIV/HBV co-infections were reported among six participants – one sample (17%) from IDUs and five (83%) from NIDUs. Triple co-infections (HBV/HCV/HIV) were detected in two patients (one from an IDU and another from NIDU). Majority of HBV/HIV co-infected were from Kisumu, that is, five samples (83%), and only one sample (17%) was from Mombasa. All HCV/HIV co-infections (100%) were detected among participants recruited from Mombasa town and majority (> 70%) were IDUs.

Among drug users studied, HBV and HCV infections were significant (*p* < 0.05) predictors of HIV infection (Table 2), partially explained by the three viruses sharing modes of transmission. The towns from which drug users were recruited and their ethnicity significantly predicted HIV infection, suggesting geographical differences in ethnic distribution and prevalence of HIV infection. Of the drug users in this study, the majority (501) were non-injectors, suggesting a prevalence of 19.3% (201) of injection drug use. HIV infection was significantly higher among injection drug users compared with non-injection drug users (*c* ^2^ = 3.39, *p* = 0.007). Age, sex, level of education and sexual debut were not significant predictors of HIV infection in this population (Table 2).

**TABLE 2:** Predictors of human immunodeficiency virus infection among drug users from three major cities in Kenya.

Characteristic	Category	*N*	*n*	%	Chi square (*χ*^2^)	*p*
Age (years)	15–19	37	4	10.8	10.388	0.065
	20–24	241	18	7.5		
	25–29	150	15	10.0		
	30–34	136	24	17.6		
	35–39	80	9	11.3		
	≥ 40	29	5	17.2		
Sex	Male	626	66	10.5	3.270	0.071
	Female	47	9	19.1		
HBV infection	Positive	29	7	24.1	5.167	0.023
	Negative	644	68	10.6		
HCV infection	Positive	44	18	40.9	42.947	0.000
	Negative	628	56	8.9		
Current town of residence	Kisumu	N-121	22	18.2	14.364	0.001
	Mombasa	305	39	12.8		
	Nairobi	247	14	5.7		
Level of education	≥ Grade 12	228	30	13.2	0.336	0.562
	< Grade 12	296	34	11.5		
Main source of income	Regular work	328	48	14.6	8.573	0.036
	Welfare	140	8	5.7		
	From friends	166	16	9.6		
	Other	22	2	9.1		
Ethnicity	Kikuyu/Kamba	88	6	6.8	9.552	0.023
	Luo	122	21	17.2		
	Luyia	68	3	4.4		
	Other	362	39	10.8		
Injected non-prescribed drug	Yes	120	19	15.8	3.392	0.007
	No	501	48	9.6		
Age at sexual debut	< 15	127	11	8.7	2.986	0.225
	15–19	344	45	13.1		
	≥ 20	179	16	8.9		

HBV, hepatitis B virus; HCV, hepatitis C virus.

Any of the three infections was detected in 18.3% (123/673) of drug users. Factors negatively associated with any infection (Table 3) included younger age (30 years old; adjusted odds ratio [aOR] = 0.50, 95% CI: 0.33–0.76; *p* < 0.001) and early sexual debut (aOR = 0.54, 95% CI: 0.40–0.82; *p* < 0.05). Younger age was also negatively associated with HIV infection (aOR = 0.53, 95% CI: 0.31–0.89; *p* < 0.01) and HCV infection (aOR = 0.29, 95% CI: 0.15–0.56; *p* < 0.001). IDU was positively associated with HCV (aOR = 5.37, 95% CI: 2.61–11.06; *p* < 0.001), while early sexual debut had negative association with HCV infection (Fisher’s Exact OR = 0.21, 95% CI: 0.07–0.58; *p* < 0.001). None of these factors were associated with HBV infection (Table 3).

**TABLE 3:** Factors associated with human immunodeficiency virus, hepatitis B virus or hepatitis C virus infections among drug users from three major cities in Kenya.

Factor	Variable	Any infection (HIV, HBV or HCV)					HIV					HCV					HBV				
		*n*	%	aOR	95% CI	*p*	*n*	%	aOR	95% CI	*p*	*n*	%	aOR	95% CI	*p*	*n*	%	aOR	95% CI	*p*
Age	≤30 years (469)	70	14.9	0.50	0.33–0.76	<0.001	43	9.2	0.53	0.31–0.89	<0.01	19	4.1	0.29	0.15–0.56	<0.001	20	4.3	0.97	0.41–2.33	0.904
	>30 years (204)	53	26.0	Reference group			32	15.7	Reference group			26	12.7	Reference group			9	4.4	Reference group		
Level of education	<12 years (445)	77	17.3	0.83	0.54–1.27	0.420	45	10.1	0.74	0.44–1.25	0.290	27	6.1	0.75	0.39–1.46	0.462	17	3.8	0.71	0.32–1.62	0.502
	≥12 years (228)	46	20.2	Reference group			30	13.2	Reference group			18	14.0	Reference group			12	5.3	Reference group		
Sexual debut	<15 years (236)	33	14.0	0.54	0.40–0.82	<0.05	24	10.2	0.86	0.50–1.47	0.644	5	2.1	0.21	0.07–0.58A	<0.001	11	4.7	1.14	0.49–2.59	0.895
	≥15 years (437)	90	20.6	Reference group			51	11.7	Reference group			40	9.2	Reference group			18	4.1	Reference group		
Drug use	IDU (120)	27	22.5	1.51	0.89–2.52	0.13	19	15.8	1.63	0.87–3.01	0.07	20	16.7	5.37	2.61–11.06	<0.001	1	0.8	0.19	0.01–1.35	0.097
	NIDU (501)	81	16.2	Reference group			48	9.6	Reference group			18	3.6	Reference group			24	4.8	Reference group		
Perceived risk of infection	Little or no risk (65)	-	-	-	-	-	9	13.8	1.32	0.58–2.91	0.606	10	15.4	1.46	0.65–3.21	0.417	7	10.8	1.65	0.62–4.24	0.301
	Somewhat or high risk (607)	-	-	-	-	-	66	10.9	Reference group			34	5.6	Reference group			21	3.5	Reference group		
Perceived unprotected sex	About half to most (176)	26	31.8	0.67	0.40–1.10	0.118	16	14.8	0.69	0.37–1.29	0.275	8	4.5	0.53	0.22–1.22	0.151	8	4.5	1.11	0.43–2.75	0.992
	Very few to none(436)	90	20.6	Reference group			55	12.6	Reference group			36	8.3	Reference group			18	4.1	Reference group		

A, Fisher’s Exact test applies; aOR (adjusted odds ratio), adjusted for city of residence, gender and ethnic group.

HIV, human immunodeficiency virus; HCV, hepatitis C virus; HBV, hepatitis B virus; CI, confidence interval.

### Hepatitis B virus genotyping results

Based on HBV serological test, 30 samples were positive for HBsAg and when subjected to genetic analysis to determine genotypes and subgenotypes, only 16 samples (53%) out of the 30 samples were assigned or classified into genotypes based on two regions (BCP and S) that were amplified to obtain genotypes and subgenotype results. Table 4 shows the distribution of HBV subgenotypes from three recruitment sites (Kisumu, Nairobi, Mombasa) in Kenya.

**TABLE 4:** Distribution of hepatitis B virus subgenotypes among drug users from three major cities in Kenya.

Participant ID. No	Gender	Age(years)	Area (town)	Nested BCP PCR	Large S PCR	Subgenotype
108	Male	20	Nairobi	+	–	A1
111	Male	30	Nairobi	+	+	A1
155	Male	21	Nairobi	+	+	A1
163	Male	23	Nairobi	+	+	D6
166	Male	22	Nairobi	+	+	Mixed
174	Male	26	Nairobi	+	+	D6
244	Male	25	Nairobi	+	+	A1
282	Male	42	Kisumu	+	–	A1
332	Male	20	Kisumu	+	+	A1
426	Female	38	Mombasa	–	+	D6
445	Male	26	Mombasa	+	+	A1
496	Male	42	Mombasa	+	+	Mixed
501	Male	38	Mombasa	+	+	A1
522	Male	23	Mombasa	–	+	A1
645	Male	28	Mombasa	+	+	A1
729	Male	30	Mombasa	+	+	Mixed

BCP, basic core promoter; PCR, polymerase chain reaction.

**FIGURE 1:**
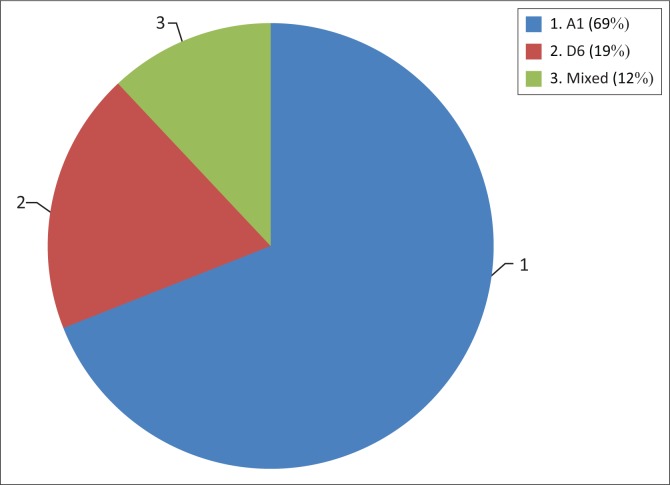
Hepatitis B virus subgenotypes isolated from Kenyan drug users.

Out of the 16 samples, 11 samples (69%) constituted the majority and were assigned subgenotype A1. Three samples (19%) were classified subgenotype D6 and two samples (13%) failed subgenotype classification (mixed population). Among the two samples that failed subgenotype classification, one came from Kisumu and another from Nairobi. Out of the 11 subgenotypes A1 isolates identified, four were from Nairobi, five from Mombasa and two from Kisumu. Among those with D6 subgenotypes, two came from Nairobi and one from Mombasa. Figure 1 shows the distribution of HBV subgenotypes. There was no significant difference between the distribution of the subgenotypes in the different towns.

### Hepatitis C virus genotyping results

Hepatitis C virus molecular amplification was performed on 39 samples based on 5’UTR and NS5B regions for genotyping and subgenotyping classification. All the 39 samples were amplified and sequenced (21 samples on 5’UTR region and 18 samples on NS5B) to assign genotypes and subgenotypes. Five out of 21 (24%) sequences were classified into genotype 1, 14 out of 21 (67%) into genotype 4 and one sample into genotype 2 (5%). Among the 18 that were subgenotyped on NS5B region, 4 out of 18 samples (22%) were classified into subgenotype 4a, 13 samples (72%) into subgenotype 1a and two samples (6%) were not subgenotyped.

## Discussion

The majority of participants (93%) in this study were male and a similar occurrence was observed among drugs users in Nairobi where male participants were in the majority compared to female participants.^7^ Infection with blood-borne viruses (HBV, HCV and HIV) presents a great public health challenge regarding the management, resource allocation and risks for co-infections owing to shared modes of transmission. This study found a higher prevalence of HIV (11.1%) compared to 6% in the general population^54^ and 4.5% HCV prevalence against 1.8% recorded among blood donor populations.^14^ However, a lower HBV prevalence of 6.5% was observed among drug users in this study compared to 13.3% reported among Nairobi urban slum dwellers.^14^ In a similar study conducted in Kisumu to determine the HBV and HCV among discordant couples irrespective of their HIV status, a prevalence of 4% and 5.6% for HCV and HBV infections, respectively, was reported.^55^ The predominance of male participants (93%) in this study was observed compared to female participants. The higher proportion of NIDUs (74%) over IDUs (26%) may account for the overall differences in the HBV prevalence among drug users. When compared, HBV prevalence was higher in Nairobi (52%), followed by Mombasa (30%) and Kisumu (17%). We do not know at present why this difference is so dramatic and further research should be considered to try and understand the difference. The HBV vaccination in Kenya under the Expanded Programme on Immunisation was launched in Kenya in 2003 and participants would not have benefited from it nor would it have resulted in HBV prevalence. A catch-up/targeted vaccination for at-risk population was not available. However, more HCV infections (93%) were concentrated in Mombasa and only 7% in Nairobi. None was reported from Kisumu. We do not know exactly why the difference in HCV prevalence was so dramatic.

The IDU population, although small, shows a higher proportion of HCV mono-infections (93%) and HCV/HIV co-infection (72%). This finding is consistent with previous studies in Kenya^29,46^ and elsewhere.^56,57^ The use of unsafe injection practices has been linked with HCV and HIV infections among drug users.^58^ In contrast, HBV/HIV co-infections were more common among NIDUs (83%) compared with IDUs (17%). This occurrence may be associated with unprotected sexual practices shown in previous studies.^59^ The two samples with triple co-infections (HBV/HCV/HIV) and those with either HBV/HIV or HCV/HIV confirm the shared routes of transmission of the viruses as previously documented (1–3).

### Hepatitis B virus genotypes and phylogenetic analysis

HBV DNA was detected in 50% of the HBsAg-positive samples (16/30). This percentage was much lower when compared to 60% reported from a previous study conducted among patients with and without liver disease in Sudan.^60^ The differences would have been owing to differences in population characteristics. Low detection of subgenotype A1 has been observed previously and thought to be associated with low viral loads. In this study, HBV genotype A (11/16; 69%) was the most prevalent, followed by D (3/16; 19%). Two samples failed subgenotype assignment and this was thought to be because of mixed infections. All A genotypes isolated belonged to subgenotype A1 while genotype D isolates belonged to subgenotype D6. The dominance of HBV subgenotype A1 is consistent with other previous studies among blood donors,^61,62^ HIV/HBV co-infected drug users,^63^ jaundiced outpatients^36^ and patients with liver disease (HCC)^36^ in Kenya and India.^64^ HBV subgenotype A1 is believed to be endemic in Africa^36^ and mainly found in the eastern, central and southern Africa.^65^ Isolation of only HBV subgenotype A1 from all the three recruitment sites (Mombasa, Nairobi and Kisumu) is consistent with its endemicity and predominance in Eastern Africa as previously reported.^60,62,64^ Although HBV subgenotype A2 has been reported among blood donors,^66^ it was found to be a mis-classification^36^, which is consistent with our study that found none despite samples obtained across three major towns in Kenya, suggesting a need for continuous molecular studies to confirm HBV genetic diversity in Kenya. HBV genotype D was detected and its subgenotype grouped into D6. This finding was consistent with an earlier study among blood donors.^36^ However, our study shows a higher percentage (19%) of HBV subgenotype D6 compared to 4% found in a previous study.^7^ This subgenotype (D6) has also been reported in Egypt,^67^ North Africa^54^ and Sudan (Khartoum).^15^

### Hepatitis C virus genotypes and subgenotypes

Phylogenetic analysis of HCV isolates revealed the presence of HCV genotypes (1,2 and 4) and two subgenotypes (1a and 4a). These results are consistent with previous studies that were conducted among blood donors^55^ and drugs users^56^ in Kenya. In this study, HCV subgenotype 1a was predominant (72%). The predominance of HCV subgenotype 1a also has been reported in West Africa^57^ and almost exclusively in North America.^58^ However, certain factors favour predominance of specific HCV genotypes and subgenotypes in different geographical locations within a country around the world. For instance, in a previous study, HCV genotype 2a was found to be predominant among the blood donor population in Kenya,^55^ whereas in this study, HCV subgenotype 1a is predominant among the population of drug users, especially the IDUs, suggesting a need for continuous monitoring of these for better treatment outcomes that entirely depend on HCV genotyping results. Similar observations were reported from a study conducted in Pakistan to determine the distribution of HCV genotypes in different geographic locations.^59^ Although our study generated tangible information on HBV and HCV molecular epidemiology among drug users, including the management of these viral infections, there were some limitations. Majority of the participants were male (93%); females may have been underrepresented or be less likely to use drugs compared to males in Kenya. Both injecting and NIDUs’ participation were not balanced and evenly distributed in each town represented in the analysis; hence, a bias in data analysis may have occurred.

## Conclusion

In conclusion, our study found HBV, HCV and HIV prevalence of 4.3%, 6.5% 11.9%, respectively, which were detected including their co-infections (HBV/HIV, HCV/HIV, HBV/HCV/HIV). Molecular analysis revealed HBV genotypes A1, D6 and mixed genotypes in circulation, and HCV subgenotypes 1a and 4a were isolated among drug users. This high level of genetic diversity exhibited by HBV and HCV isolates requires continuous monitoring for effective management of the patients and full implementation of harm reduction strategies to reduce mortality and morbidity associated with these viral infections.
